# Phenotypical characterization, and antibiotics susceptibility patterns of skin bacteria found in podoconiosis patients in the North West Region of Cameroon

**DOI:** 10.1186/s12866-023-02923-9

**Published:** 2023-07-17

**Authors:** Derick Lekealem Nkwetta, Bangsi Rose Fuen, Njodzeka Flora Yenban, Nancielle Mbiatong, Gordon Takop Nchanji, John Bonekeh, Bertrand Lontum Ndzeshang, Narcisse Victor Tchamatchoua Gandjui, Fanny Fri. Fombad, Ute Klarmann-Schulz, Mathias Eyong Esum, Abdel Jelil Njouendou, Jerome Fru Cho, Achim Hoerauf, Manuel Ritter, Samuel Wanji

**Affiliations:** 1 Department of Microbiology and Parasitology, University of Buea, P.O. Box 63, Buea, Cameroon; 2 Research Foundation for Tropical Diseases and the Environment (REFOTDE), P.O. Box 474, Buea, Cameroon; 3 The Peoples’ Hope Medical Care Centre (PHOMECC), Bamenda, Cameroon; 4 Institute for Medical Microbiology, Immunology and Parasitology (IMMIP), University Hospital Bonn (UKB), Bonn, Germany; 5 German Centre for Infection Research (DZIF), Neglected Tropical Disease, partner site, Bonn- Cologne, Bonn, Germany; 6 Department of Biomedical Sciences, University of Buea, P.O. Box 63, Buea, Cameroon; 7 German-West African Centre for Global Health and Pandemic Prevention (G-WAC), Partner Site, Bonn, Bonn, Germany

**Keywords:** Antibiotic, Susceptibility, Podoconiosis, Skin, bacteria, Phenotypic

## Abstract

**Background:**

Podoconiosis, a non-infectious disease originating from long-term exposure of bare feet to irritant red clay soil is a lifelong, disabling disease with no specific diagnostic tool, classified into 5 stages based on the severity of leg swelling (lymphoedema). Secondary bacterial infections have been suggested to cause acute dermatolymphangioadenitis (ADLA) attacks and drive disease progression. Although the North West Region of Cameroon has a proven history of podoconiosis endemicity, the bacterial composition of lymphoedema due to this condition has not been studied. Thus, this study investigated the leg bacterial diversity of patients who suffered from the lymphoedema and their susceptibility pattern to selected antibiotics.

**Methods:**

A cross-sectional study was carried out in which podoconiosis affected and non-lymphoedema individuals living in the same community were purposively selected. Samples were collected by swabbing the skin between the toes and around the anklebone, then cultured and sub-cultured on nutrient agar to obtain pure isolates. The cultured isolates were then morphologically and biochemically classified using microscopy and analytic profile index test kits, respectively. The disk diffusion technique was used to determine antibiotic susceptibility.

**Results:**

Thirty-three participants were recruited, and 249 bacterial isolates were characterized into 29 genera, 60 species; with 30 (50%) being gram positive rods, 19 (31.7%) gram positive cocci, and 11 (18.3%) gram negative rods. Thirteen gram positive rods, fifteen gram positive cocci, and eight gram negative rods of bacterial species were found only in podoconiosis individuals among which *Cellulomonas spp / Microbacterium* spp. (2.8%), *Staphylococcus lentus* (3.3%), and *Burkholderia cepacia* (4.0%) dominated. 90% (90%) of the bacterial isolates were sensitive to doxycycline, whereas ampicillin had a high level of intermediate resistance, and penicillin G had the greatest resistant profile.

**Conclusion:**

Our findings show that 94 (37.8%) out of 249 described bacterial isolates were exclusively found in the legs of podoconiosis individuals, and their susceptibility pattern to antibiotics was similar to that of others.

**Supplementary Information:**

The online version contains supplementary material available at 10.1186/s12866-023-02923-9.

## Introduction

Podoconiosis is a non-infectious disease originating from long term exposure of barefoot to irritant red clay soil in areas of high altitude [1]. The term podoconiosis, established by Price in the 1970s, derived from the Greek words “*podos*” and “*konio*” which mean “foot” and “dust”, respectively [2]. Globally, podoconiosis affects an estimated 4 million people in over 32 countries, 18 of which are in Africa, with highest prevalence values reported in Tanzania (2.5%), Kenya (3.9%), Uganda (4.5%), Ethiopia (7.5%), and Cameroon (8.1%) [3, 4]. In 2017, at least one case of podoconiosis was reported in each of the 10 Regions in Cameroon and the highest prevalence (1.7%) was in the North West Region, followed by the North Region with 1.0% [5]. The disease is asymptomatic in the early teens and can affect several individuals from the same family, and nocturnal leg and foot pain is a classic feature in young person’s [6]. Symptoms usually start with itching and a burning sensation, followed by a sign such as swelling in the sole [2]. The disease is characterised by massive bilateral asymmetrical swelling of the lower legs and feet [7] and is classified into 5 stages based on the severity of the swelling [8]. Thus, podoconiosis-driven lymphedema and consequently elephantiasis is a developmental challenge since it affects the active population, thereby reducing productivity and bringing about disability-adjusted life years [9]. Thus, early, and accurate diagnosis of lymphoedema is very important as it significantly increases the success of treatment and prevention [10]. This corroborates findings from three studies that have reported podoconiosis to be curable at the pre-elephantiasis phase [11-13] and that hygiene-based management, using soap, clean water, and antiseptics, significantly improves the morbidity of the disease [14, 15]. In addition, prevention has been shown to be achieved by avoiding direct contact of bare feet with irritant red clay soil [16].

To date, there is no recommended diagnostic test for podoconiosis, though clinical examination and medical history found in the algorithm of podoconiosis management remain the corner stone of diagnosis [9]. This poses a major challenge not just in the case of the management of the disease but also in understanding the etiology, epidemiology, and disease burden in endemic areas [17]. Currently, podoconiosis can only be screened clinically by excluding hereditary, as well as infectious causes of lymphoedema [18], including lymphatic filariasis (LF) and leprosy, must be distinguished during physical examination and clinical history [19]. Despite the availability of treatment and management options for other forms of lower limb lymphoedema (such as LF and leprosy), there is no treatment for podoconiosis. It is hypothesised that the disease develops when mineral particles of elements (such as aluminium, silicon, magnesium, and iron) common in clay soils penetrate the sole of the leg and are taken up by macrophages into the lymphatic system, leading to an inflammatory response [20]. However, the distinct source and mechanism of disease progression remain uncertain. Most individuals suffering from podoconiosis do not know the best way to care for their legs, thereby accumulating dirt, which may create a new microenvironment favourable for colonization by microbes, leading to worsening of the disease [21]. To support this fact, it has been shown that patients with lymphoedema are susceptible to cellulitis, which is mainly caused by group A *Streptococcus* [22]. In addition, secondary bacterial and/or fungal infections have been suggested as a major outcome of podoconiosis [23]. Despite the fact that podoconiosis is not classified as an infectious disease, inflammatory episodes, called acute dermatolymphangioadenitis (ADLA) attacks have been found to be associated with the penetration of bacteria into the dry cracked skin on the feet, which cause inflammation and the development of lymphoedema [24–26], similar to what occurs in LF [27–30]. ADLA is one of the most serious complications of podoconiosis, and the dermatological life quality index (DLQI) in podoconiosis is like LF and has been associated with ADLA [31, 32]. Thus, it is strongly thought that microbe’s may be important contributing elements driving lymphoedema progression due to podoconiosis, but the bacterial composition of podoconiosis has not yet been characterised. Therefore, this study characterised the bacterial composition on podoconiosis-driven lymphoedema legs and determined their susceptibility pattern to selected antibiotics in the North West Region (NWR) of Cameroon.

## Methods

### Study area

This study was conducted at the Bamenda Clinical Trial Centre (BCTC), or Podoconiosis Treatment Centre, situated at Foncha Street Nkwen, Bamenda, head-quarter of the NWR of Cameroon, within the TAKeOFF/LE-doxy trial. The presence of podoconiosis had previously been demonstrated in the NWR by Deribe et al., during a nationwide mapping campaign in 2018 [3], highlighting it as the region with the highest prevalence of podoconiosis among the 10 regions of the country. The NWR is composed of mostly hilly land with a mean altitude of 1403 m above sea level. It has two seasons, the dry and the wet, with an average annual rainfall of 2500 mm^3^. Because of the extremely fertile soil of this region which favours the growth of a variety of cash crops and other vegetables, farming is the primary occupation of the inhabitants [33].

### Study design and sampling method

A cross-sectional study was conducted in which podoconiosis patients with one or both affected legs and non-lymphoedema control individuals living in the same communities as the patients visiting the BCTC or Podoconiosis Treatment Centre were purposively selected. The eligible participants who agreed to participate in the study were given information about the background and objectives of the study, and the possible benefits and discomforts that may occur with their involvement.

### Study population and selection criteria

The study population consisted of clinically confirmed males or non-pregnant females with podoconiosis of stages 1 to 5, classified according to the *de novo* clinical staging system by Tekola and colleagues [8], and controls, who reside in the same area but do not have lymphedema. All study participants were 18 years of age and older and had lived in the NWR for at least 2 years. Those who had severe or systematic co-morbidities were not included. Each participant signed or provided a thumbprint on informed consent forms.

### Laboratory procedures

#### Sample collection

Following identification of eligible individuals who visited the BCTC, a questionnaire-based survey was used to collect their socio-demographic data like gender, age, occupation, duration in the community, educational level, and podoconiosis stages (Additional file 1). Individual codes were generated and assigned to each participant who had given consent, then used throughout the study. Samples were collected from both legs of each participant using separate, pre-labelled sterile cotton swabs (Jeanne, LOT: 200,615) by swabbing the skin areas between toes, in skin folds when applicable, and around the anklebone following rigorous aseptic techniques to avoid contamination.

#### Culture and pure culture isolation

Samples were inoculated and sub-cultured based on their morphological presentations on prepared nutrient agar (Oxoid Ltd, Basingstoke, UK, REF: CM0003) plates by quadrant streaking and incubated at 37^o^C for 24 h to obtain pure isolates. Each pure colony was allocated a code and serial number. Pure isolates were characterized phenotypically and stored in peptone water containing 15% glycerol at -20^o^C.

#### Morphological characterization of bacterial cultures and isolates

Cultures were examined macroscopically for colony colour, colony texture, elevation, shape, and margin, while stained smears of the isolates were examined microscopically for the gram reaction, shape, arrangement, and position of spores using a Humanscope light microscope (Human, City, Germany) under the 40x and 100x objectives, respectively. The motility test was carried out for gram positive and negative rod-shaped bacterial isolates using the 40x objective.

#### Biochemical characterisation of bacteria isolates using the Analytic Profile Index (API)

To biochemically differentiate those bacteria that produce the enzyme catalase, such as *staphylococci*, from non-catalase producing bacteria, such as *streptococci*, the catalase test was performed as previously described [34].

The, oxidase test strips (Biolife Italiana Srl, Milan, Italy) were used according to the manufacturer’s instructions to assist in the identification of *Enterobacteriaceae* and non-*Enterobacteriaceae* bacteria, which all produce the enzyme cytochrome oxidase.

Carbohydrate fermentation and hydrogen sulphide production tests were performed on gram negative rods cultured on Kliger iron agar (Oxoid Ltd, Basingstoke, UK) to exclude/include bacterial isolates of the family *Enterobacteriaceae*. Manitol Salt Agar (MSA) Tests using Manitol salt agar (Oxoid Ltd) to culture gram positive cocci to decipher salt tolerance grows (e.g. *Staphylococcus* spp.), which is indicated by the conversion of phenol red to yellow in comparison to salt intolerance gram positive cocci (e.g. *Streptococci* spp.). Finally, Analytical Profile Index (API) tests were used to discriminate among *Bacillus* spp. (API 50 CH, REF: 50,300), *Staphylococcus* spp. (API STAPH, REF: 20,500), *Streptococcus* spp. (API STREP, REF: 20,600), *Corynebacterium* spp. (API Coryne, REF: 20,900), *Enterobacteriaceae* (API 20 E, REF: 20,160) and non-*Enterobacteriaceae* (API 20 NE, REF: 20,050) according to the manufacture. API test kits and reagents were purchased from BioMerieux SA, Marcy-I’Etoile, France.

#### Susceptibility of bacterial isolates to antimicrobial agents

The antimicrobial susceptibility test was performed using the Kirby Bauer disk diffusion method, following the norms set by the Clinical Laboratory Standard Institute (CLSI) guidelines [35]. The antibiotic-impregnated discs were selected based on their classes, spectrum of activity, topical usages, and availability in the study area. The antibiotics used were Ampicillin-10 µg (AM-10), Amoxicillin-10 µg (AX-10), Penicillin G-10U (P-10), Ceftriazone-5 µg (CRO-5), Cefuroxime-5 µg (CXM-5), Nalidixic acid-30 µg (NA-30), Ofloxacin-5 µg (OFX-5), Nitrofurantoin-300 µg (F-300), Trimethoprim/Sulfamethoxazole 1.25/23.75 µg (SXT-25), Doxycycline-30 µg (DO-30), Tetracycline-30 µg (TE-30), Gentamycin-30 µg (CN-30), Erthromycin-15 µg (E-15), and Vancomycin-30 µg (VA-30).All antibiotics were obtained from Bioanalyse, Yenimahalle-Ankara, Turkey.

### Data Processing

Data was registered into a logbook and further entered into Microsoft Excel 2016 for descriptive analysis. Results were presented using frequency tables and bar charts.

## Results

### Socio-demographic characteristics and stage distribution

Thirty-three (33) participants were enrolled in the study, comprising 26 podoconiosis cases and 7 controls. The distribution of participants according to their socio-demographic characteristics and lymphedema stages is shown in Table 1. More than 2/3 of the total participants were females (75.8%). Their mean age was 43 years (range: 18–60) and most of them were farmers (36.4%). Main occupation was farming Twenty-seven (81.8%) of the participants had lived within the endemic community, for more than 20 years. Five (15.2%) participants had no education. This study had seven stage 0, six and seven stages 2 and 3 participants respectively, who had bilateral symmetrical podoconiosis, and the rest of the participants presented with bilateral asymmetrical podoconiosis (Table 1).

### Description of the characterized bacterial populations

A total of 249 isolates, including 215 from podoconiosis, and 34 from controls, were identified upon culture (Table 2). Apart from the calculations in Table 2, all calculations concerning bacterial isolates are based on 249 (the total number of isolates identified).

**Table 1:** Socio-demographic characteristics and stage distribution

Variable	Class	Frequency	Percentage
Gender	Female	25	75.8
	Male	8	24.2
Age (years)	18–30	9	27.3
	31–40	7	21.2
	41–50	5	15.2
	51–60	12	36.3
Occupation	Business	2	6.1
	Farmer	12	36.4
	Farmer/business	1	3
	Hair dresser	2	6.1
	Nurse	2	6.1
	Seamstress	2	6.1
	Student	4	12.1
	Others	8	24.1
Duration in the community (Years)	2–10	1	3.0
	11–20	5	15.2
	21–30	7	21.2
	31–40	7	21.2
	41–50	4	12.1
	51–60	9	27.3
Years of education	0	5	15.1
	1–7	12	36.4
	8–15	12	36.4
	16–22	4	12.1
Stage distribution of participants	Stage 0 or controls	7	21.3
	Stage 2 both legs	6	18.2
	Stage 3 both legs	7	21.3
	L0R3	3	9.0
	L1R4	1	3.0
	L2R0	1	3.0
	L3R0	2	6.1
	L3R4	2	6.1
	L4R2	1	3.0
	L4R3	1	3.0
	L4R4	1	3.0
	L4R5	1	3.0

**L = Left leg, R = Right leg.**

**Table 2:** Description of bacterial population isolated from participants

Gram presentation	Isolates from Podoconiosis n (%)	Isolates from Controls n (%)	Isolates from both groups n (%)	Isolates from podoconiosis only n (%)	Isolates from controls only n (%)
**G** ^**+**^ **cocci**	88 (40.9)	8 (23.5)	69 (46.6)	27 (29.7)	0 (0.0)
**G** ^**+**^ **rods**	92 (42.8)	24 (70.6)	79 (53.4)	29 (31.8)	8 (80.0)
**G** ^**−**^ **rods**	35 (16.3)	2 (5.9)	0 (0.0)	35 (38.5)	2 (20.0)
**Total**	215 (100.0)	34 (100.0)	148 (100.0)	91 (100.0)	10 (100.0)

n = number of isolates, G^+^ = gram positive and G^−^ = gram negative. Isolate from Podoconiosis: number of various isolates that were analyzed from all podoconiosis individuals, ragrless of their presence in control group. Isolates from Controls: number of various isolates that were analyzed from all controls, regardless of their presence in podoconiosis individuals. Isolates from both groups: number of various isolates that were found in podoconiosis and control groups. Isolates from podoconiosis only: number of various isolates that were found in podoconiosis cases but not found in control group. Isolates from controls only: number of various isolates that were found only in control, but not found in podoconiosis group.

**Fig. 1:**
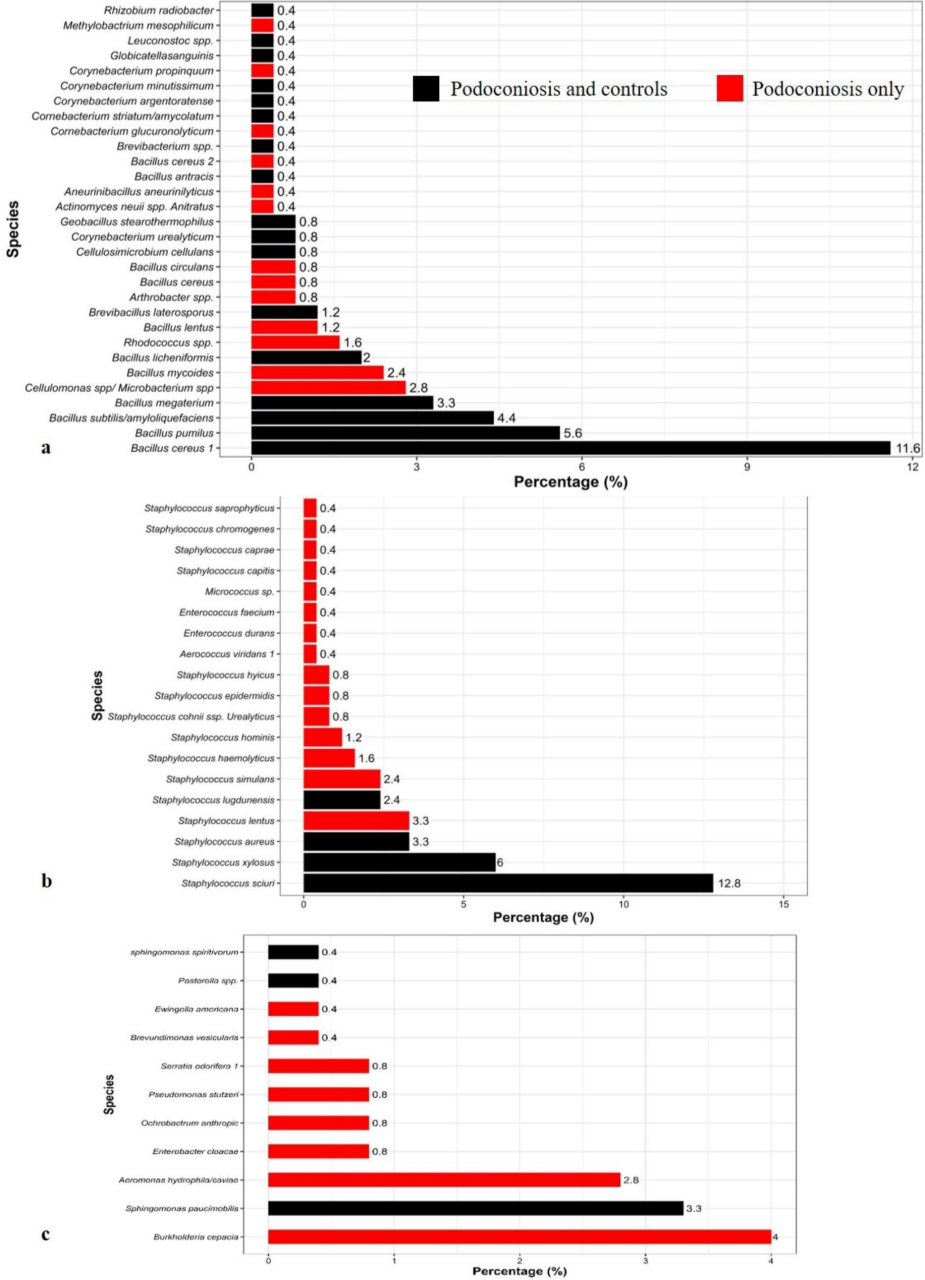
Prevalence of bacterial population isolated from study participants. a: gram positive rods, b: gram positive cocci, c: gram negative rods

In total, 116 (46.6%) gram positive rods, 96 (38.5%) gram positive cocci and 37 (14.9%) gram negative rods were identified from the bacteria cultures of podoconiosis individuals and controls. A total of sixty (60) species were identified, consisting of 30 (50%) gram positive rods, 19 (31.7%) gram positive cocci and 11 (18.3%) gram negative rodsThirteen gram positive rod species, fifteen species of gram positive cocci, and eight species of gram negative rods were isolated only from podoconiosis patients, among which *Cellulomonas spp / Microbacterium* spp. (2.8%), *Staphylococcus lentus* (3.3%), and *Burkholderia cepacia* (4.0%) dominated respectively (Fig. 1).

### Podoconiosis stage-specific distribution of bacterial species

To investigate which leg stages harbour distinct bacteria spp., we compared their composition on leg stages 2 − 5 and observed that A*rthrobacter* spp. (0.8%), *Actinomyces neuii* ssp. *Anitratus* (0.4%), *Bacillus cereus* 2 (0.4%) and *Enterococcus faecium* spp. (0.4%) were specific for stage 2 legs. Stage 3 legs specifically harbour *Serratia odorifera* 1, *Staphylococcus cohnii* ssp. *Urealyticus*, and *Staphylococcus epidermidis* (0.8% each), followed by *Aerococcus viridans* 1, *Brevundimonas vesicularis*, *Cornebacterium glucuronolyticum, Ewingella Americana, Methylobactrium mesophilicum, Micrococcus* sp., *Staphylococcus capitis*, *Staphylococcus chromogenes*, and *Staphylococcus saprophyticus* (0.4% each). Finally, on stage 4, we specifically identified three distinct bacteria species, including *Aneurinibacillus aneurinilyticus*, *Corynebacterium propinquum*, and *Staphylococcus caprae* (0.4% each), whereas for stage 5, only *Enterococcus durans* (0.4%) could be specifically identified.

### Antibiotic susceptibility profile of characterized bacteria species

**Fig. 2:**
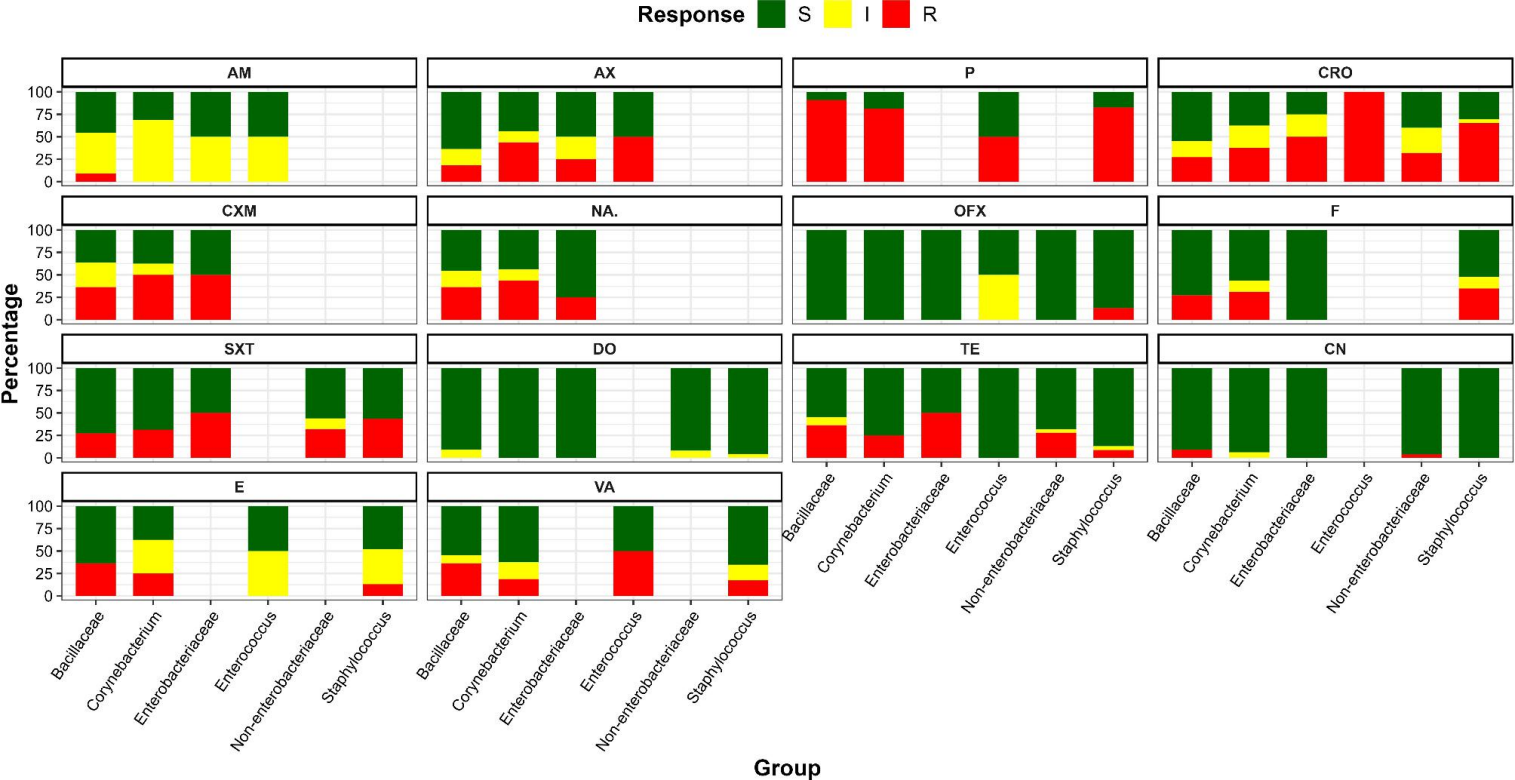
Antibiotic susceptibility profile of bacteria groups.(AM; Ampicillin, AX; Amoxicillin, P; Penicillin G, CRO; Ceftriazone, CXM; Cefuroxime, NA; Nalidixic acid, OFX; Ofloxacin, F; Nitrofurantoin, SXT; Trimethoprim/Sulfamethoxazole, DO; Doxycycline, TE; Tetracycline, CN; Gentamycin, E; Erthromycin, and VA; Vancomycin)

To test the antibiotics susceptibility of the obtained isolates, the Kirby Bauer disk diffusion method was applied, which revealed that most of the bacteria were sensitive to Doxycycline, Gentamycin, and Ofloxacin. A high rate of intermediate was recorded with Ampicillin while high resistance was recorded with Penicillin G. Bacteria species that were specific to podoconiosis did not exhibit different antibiotic pattern to those that were not (Fig. 2).

## Discussion

Despite the fact that podoconiosis causes lifelong disability, stigmatization, and reduces economic productivity, especially in the agricultural sector, recognizing podoconiosis as an important public health concern remains a challenge [36]. The high proportion of females suffering from podoconiosis-driven leg lymphoedema, with farming being their most predominant occupation, is in line with research conducted by Wanji et al. [37] in the NWR of Cameroon, which is probably due to their high involvement in bare feet agricultural activity. Here, the majority 17 (> 51.6%) of participants were aged 40 years and above, with similar observations by Deribe et al. [3]. during the nationwide mapping of podoconiosis in Cameroon, and this is because the disease is asymptomatic in the early decades of life. A good number (26) of our study participants had lived in an endemic area for more than 20 years, confirming previous studies showing that duration in the community is an important factor to detect podoconiosis [1].

In regards to the composition of bacteria spp. on the skin of lymphedema legs, we could observe that *Staphylococcus* (36.9%) dominated, which is in line with the work by Grice and Serge [38] and Costello et al. [39]., who reported it as one that dominates in moist areas (toe web) of the skin with super-antigens responsible for recurrent skin inflammation. Indeed, *Staphylococcus* and *Bacillus* have been isolated as one of the bacteria that provoke ADLA in lymphatic filariasis [29], which is also a serious clinical manifestation of podoconiosis, suggesting that *Staphylococcus* and *Bacillus species* could possibly provoke the worsening of lymphoedema and disease progression. In addition, as the third most dominant genus, *Burkholderia* is a significant resident bacterium in soil and water, causing muscle or joint pain, localized swelling, skin infection and respiratory tract infection [40, 41]. Moreover, *Burkholderia* was not found in control legs, confirming that it is not present on normal skin microbiota [38] and thus, may be implicated in podoconiosis since the leg is compromised [8]. In addition, we also obtained *Staphylococcus sciuri* (12.8%) on lymphoedema legs, which is mostly associated with soil and water and could be isolated from a variety of animals [42]. It rarely causes infection, though a case report has shown that it causes inflammation and wound infections [43]. These are also some of the features of podoconiosis that participants expressed, especially wound infections in the late stage of the disease. *Staphylococcus xylosu* was the second most abundant gram-positive coccus *s* (6%). This is a commensal bacterium that rarely causes infection though it has been reported to cause redness (erythema) and skin lesions on the legs [44], with clinical presentations similar to those of ADLA caused by secondary bacteria [27, 45]. Moreover, *Staphylococcus lentus* has been reported to rarely cause disease, though it has been reported to cause peritonitis, which is a form of inflammation of the peritoneum [46]. *Staphylococcus aureus* is a bacterial specie reported to cause a lot of diseases, most especially skin lesions such as inter-digital lesions in toe webs [47] and cellulitis. Indeed, podoconiosis disease also shows cracks or breaches in the skin in the late form of the disease that gives an entry portal for such bacteria [24, 48]. Toe-web lesions a clinical manifestation of podoconiosis, particularly during an ADLA attack, and *Staphylococcus simulans* has been identified as an emerging pathogen of skin and soft tissue infection [49]. Also, *Staphylococcus lugdunensis* has been reported to be a culprit in skin and soft tissue infection [50]. Given that podoconiosis develops from soft tissue to hard keratinised forms [51], it is reasonable to believe that the soft or fluid forms may facilitate colonization by these bacteria species, which are not harmful to healthy legs [16].

With respect to the gram-positive rods found in the study, *Bacillus cereus* 1 showed a high relative abundance (11.6%). Although this bacterial specie is not a frequent causative agent of cutaneous infection, we are bound to have a high index of suspicion in certain circumstances due to its high distribution in the environment. Indeed, it has been reported that patients with compromised skin are vulnerable to cutaneous infection by *Bacillus cereus* [52]. In the case of podoconiosis, the legs are compromised by moss formation, folding, and wounds, and this may pave a good path for these bacteria to enter and cause more damage. *Bacillus pumilus* is a rare opportunistic pathogen that has been link to sepsis and the severity of LF in a case study [29, 53]. Thus, the identification of these bacteria in podoconiosis may possibly indicate their participation in the severity of the disease. Also, *Bacillus megaterium* is a bacterium that rarely causes disease but has been reported to infiltrate injured wounds and cause disease [54]. Since higher stages of podoconiosis often appear with present wounds [8], it is possible that this bacterium may infiltrate these wounds, thereby worsening the state of the disease. Moreover, *Cellulomonas/Microbacterium* causes infections in immune-compromised patients [55]; in podoconiosis, the legs are compromised, which may facilitate bacterial colonization [14]. Although *Bacillus mycoides* has been reported to be non-pathogenic, a case report of the frequent isolation of spores in blood incriminated the organism as a cause of bacteraemia [56], which is also a clinical presentation of podoconiosis. Therefore, the isolation of this bacterial specie in our participants is an indication that it may play a role in the development of podoconiosis. In addition, *Bacillus licheniformis* is a bacterium that is increasingly being recognized as a human pathogen and causes serious infections, including sepsis, mainly in immune-compromised patients [57].

In regards to the gram negative rods found in the study, *Burkholderia cepacia* was identified in 30.3% of our study participants making up 4.0% of the total bacteria isolated. It was identified only in podoconiosis participants and is not present in the normal flora of the skin. This bacterium has been reported to cause serious respiratory tract infections, bacteraemia, peritonitis and even spontaneous septic arthritis [58]. The demonstration of inflammatory properties in arthritis coupled with the fact that it is not reported as a normal flora of the skin suggests that this bacterium may be playing a key role in increasing the severity and progression of podoconiosis.

In addition, *Sphingomonas paucimobilis* is an opportunistic pathogen that has been isolated from wounds, sputum, and blood [59], which may easily affect the compromised legs in podoconiosis and cause further damage to the leg. Furthermore, *Aeromonas hydrophila/caviae* can cause serious wound infection that progresses rapidly [60], whereas *Pseudomonas stutzeri* is a low virulent pathogenic bacterium that infects wounds, soft tissue and causes endocarditis [61]. *Ochrobactrum anthropi* and *Enterobacter cloacea* colonize the respiratory tract and wound and subsequently cause a variety of opportunistic infections such as endocarditis, septic arthritis, osteomyelitis, and peritonitis. More so, *Enterobacter* has been reported to colonized legs and causes infections [62]. Indeed, this bacterium was obtained from podoconiosis legs and it has been shown that Enterobacter might be able to penetrate the skin, especially in the presence of predisposition factors, and migrate into the lymph node, which contained lypmphatic fluid rich in nutrient favourable to growth [4].

Bacterial species specific to podoconiosis participants were *Burkholderia cepacia*, *Cellulomonas/Microbacterium*, *Aeromonas hydrophila/carviae*, *Bacillus mycoides*, *Staphylococcus simulans*, *Staphylococcus haemolyticus, and Staphylococcus hominis*. Most of these bacterial have been reported to be opportunistic pathogens which can cause inflammation and wound infection; and thus, these bacteria may play an important role in the worsening of lymphoedema due to podoconiosis. In addition, more than 90% of the bacteria isolates were sensitive to doxycycline, gentamycin, and ofloxacin, and those specific to podoconiosis did not show a different pattern of susceptibility. Gentamycin and doxycycline are broad-spectrum antibiotics that have a strong bactericidal and bacteriostatic effect, respectively [63], whereas ofloxacin is not a broad-spectrum antibiotic but has a bacteriostatic property [64].

### Limitations

Although, we assessed the bacteria diversity and could associate distinct bacteria and their antibiotic susceptibility patterns of individuals that suffer from lympheodema due to podoconiosis, we could not analyse the full spectrum of bacteria since anaerobic cultures could not be performed due to the missing equipment. Moreover, detailed analysis of bacteria isolates like Streptococcus or Corynebacterium spp. was not possible, since cultivation of these bacteria requires enriched media which was also not available during this initial study. However, we aim to analyse bacteria patterns on podoconiosis legs using next generation 16s rRNA gene sequencing to get a detailed picture of the bacteria diversity and associate distinct bacterial taxa with lymphedema stages.

## Conclusions

This study identified 94 (37.8%) bacterial isolates out of 249 characterized phenotypically belonging to thirty-six (36) species to be specific to podoconiosis. Even though, their susceptibility pattern to antibiotics was not exceptional, these findings support the hypothesis of contribution of microbial species in the severity of podoconiosis lymphedema. However, large scale molecular characterization of microbiodata of these patients including both bacteria and fungi, as well as their response to morbidity management might trough more light on their possible contribution to disease progression, and provides basis for improve diagnosis and management of podoconiosis-driven lymphoedema podoconiosis-driven lymphoedema.

## Electronic supplementary material

Below is the link to the electronic supplementary material.

Supplementary Material 1

## Data Availability

The datasets used and/or analysed during the current study is available from the corresponding author on request.

## Abbreviations

ADLA: acute dermatolymphangioadenitis
AST: antifungal susceptibility testing
BCTC: Bamenda clinical trial centre
CLSI: clinical laboratory standards institute
LF: lymphatic filariasis
NWR: North West Region
NA: Nutrient Agar and Spp.:Species

## References

[1] Price EW (1976). The association of endemic elephantiasis of the lower legs in East Africa with soil derived from volcanic rocks. Trans R Soc Trop Med Hyg.

[2] Price EW (1984). Pre-elephantiasic stage of endemic Nonfilarial Elephantiasis of Lower Legs: “Podoconiosis. Trop Doct.

[3] Deribe K, Cano J, Trueba ML, Newport MJ, Davey G (2018). Global epidemiology of podoconiosis: a systematic review. PLoS Negl Trop Dis.

[4] Wanji S, Deribe K, Minich J, Debrah AY, Kalinga A, Kroidl I, Luguet A, Achim H, Manuel R (2021). Podoconiosis – from known to unknown obstacles to tackle. Acta Trop.

[5] Deribe K, Beng AA, Cano J, Njouendo AJ, Fru-Cho J, Awah AR, Eyong M, Ndongmo P, Giorgi E, Pigott D, Golding N, Pullan R, Noor A, Enquselassie F, Murray C, Brooker S, Hay S, Enyong P, Newport M, Wanji S, Davey G (2017). Mapping the geographical distribution of podoconiosis in Cameroon using parasitological, serological, and clinical evidence to exclude other causes of lymphedema. PLoS Neglected Tropical Disease.

[6] Price EW (1983). Endemic elephantiasis: early signs and symptoms, and control. Ethiop Med J.

[7] Davey G, Tekola F, Newport MJ (2007). Podoconiosis: non-infectious geochemical elephantiasis. Trans R Soc Trop Med Hyg.

[8] Tekola F, Ayele Z, Mariam DH, Fuller C, Davey G (2008). Development and testing of a de novo clinical staging system for podoconiosis (endemic non-filarial elephantiasis). Trop Med Int Health.

[9] Deribe K, Nebiyu N, Melanie JN, Gail D, Hugo CT (2020). The Health and Economic Burden of Podoconiosis in Ethiopia. Trop Med Hygiene.

[10] Yüksel A, Orçun G, Yusuf V, Gencehan K, Sefa Ş (2016). Manage lymphoedema Vasa.

[11] Abrahams PW (2002). Soils: their implications to human health. Sci Total Environ.

[12] Korevaar DA, Visser BJ (2012). Podoconiosis, a neglected tropical disease. Netherland the Journal of Medicine.

[13] Deribe K, Tomczyk S, Mousley E, Tamiru A, Davey G (2013). Stigma towards a neglected Tropical Disease: Felt and enacted Stigma Scores among Podoconiosis Patients in Northern Ethiopia. BioMedical Cent Public Health.

[14] Chandler DJ, Grijsen ML, Fuller LC (2020). With bare feet in the soil podoconiosis a neglected cause of tropical lymphoedema. Dermatology.

[15] Negussie H, Molla M, Ngari M, Berkley JA, Kivaya E, Njuguna P, Davey G (2018). Lymphoedema management to prevent acute dermatolymphangioadenitis in podoconiosis in northern Ethiopia (GoLBeT): a pragmatic randomised controlled trial. The Lancet Global Health.

[16] Nenoff P, Simon JC, Muylowa GK, Davey G (2010). Podoconiosis – non-filarial geochemical elephantiasis – a neglected tropical disease?. JDDG: J Der Deutschen Dermatologischen Gesellschaft.

[17] Yotsu R, Rie. (2018). Integrated Management of Skin in NTDs-Lessions Learned from Existing Practice and Field Research. *Tropiocal Medicine and Infectious Disease* 2018, 3. 10.3390/tropicalmed3040120 PMC6306929 30441754

[18] Deribe K, Brooker SJ, Pullan RL, Sime H, Gebretsadik A, Assefa A, Kebede A, Hailu A, Rebollo MP, Shafi, Bockarie MJ, Aseffa A, Reithinger R, Cano J, Enquselassie F, Newport MJ, Davey G (2015). Epidemiology and individual, household and geographical risk factors of podoconiosis in Ethiopia: results from the first nationwide mapping. Am J Trop Med Hygiene.

[19] Davey G, GebreHanna E, Adeyemo A, Rotimi C, Newport M, Desta K. Podoconiosis: a tropical model for gene–environment interactions? Trans R Soc Trop Med Hyg 2007, 101. 10.1016/j.trstmh.2006.05.002 16884751

[20] Deribe K, Tomczyk S, Mousley E, Tamiru A, Davey G (2013). Stigma towards a neglected Tropical Disease: Felt and enacted Stigma Scores among Podoconiosis Patients in Northern Ethiopia. BioMedical Cent Public Health.

[21] Chen YE, Fischbach MA, Belkaid Y (2018). Skin microbiota–host interactions. Nature.

[22] Grada PA (2017). Lymphedema Pathophysiology and clinical manifestations.pdf. J Am Acad Dermatology.

[23] Forstner F. *Podoconiosis DermNet New Zealand* 2017. Viewed on the 20/10/2019 https://dermnetnz.org/topics/podoconiosis/.

[24] Ferguson JS, Yeshanehe WE, Matts PJ, Davey G, Mortimer PS, Fuller LC (2013). Assessment of skin barrier function in podoconiosis: measurement of stratum corneum hydration and transepidermal water loss. Br J Dermatol.

[25] Brooks J, Ersser SJ, Cowdell F, Gardiner E, Mengistu A, Matts PJ (2017). A randomized controlled trial to evaluate the effect of a new skincare regimen on skin barrier function in those with podoconiosis in Ethiopia. Br J Dermatoogyl.

[26] Phillips C, Samuel A, Tiruneh G, Deribe K, Davey G (2019). The impact of acute adenolymphangitis in podoconiosis on caregivers: a case study in Wayu Tuka Woreda, Oromia, Western Ethiopia. “If she was healthy, I would be free. PLoS Negected Tropical Disease.

[27] Goel TC, Goel A. Acute Dermatolymphangioadenitis In: *Lymphatic Filariasis* (1st Edition). Springer Singapore 2016, 133–136.

[28] Shenoy RK (2008). Clinical and pathological aspects of filarial lymphedema and its management. Korean J Parasitol.

[29] Olszewski WL, Jamal S, Manokaran G, Pani S, Kumaraswami VU, Lukomska B, Tripathi FM, Swoboda E, Meisel-Mikolajczyk F, Stelmach E, Zaleska M (1999). Bacteriological studies of blood, tissue fluid, lymph and lymph nodes in patients with acute dermatolymphangioadenitis (DLA) in course of filarial lymphedema. Acta Trop.

[30] Partono F. The spectrum of disease in lymphatic filariasis. *Ciba Foundation Symposium* 1987, 127:15–31. 10.1002/9780470513446.ch3 3297555

[31] Henok L, Davey G (2008). Validation of the Dermatology Life Quality Index among patients with podoconiosis in Southetrn Ethiopia. Br J Dermatol.

[32] McPherson T, Penzer R (2003). A comparison of quality of life and disease severity in 54 patients with lymphoedema in Guyana. Br J Dermatol.

[33] Wanji S, Kengne-Ouafo JA, Datchoua-Poutcheu FR, Njouendou AJ, Tayong DB, Sofeu-Feugaing DD, Amvongo-Adjia N, Fovennso BA, Longang-Tchounkeu YF, Tekola-Ayele F, Enyong PA, Newport MJ, Davey G. Detecting and staging podoconiosis cases in NWR Cameroon: positive predictive value of clinical screening of patients by community health workers and researchers. *BioMed* Cent Public Health 2016, 16. 10.1186/s12889-016-3669-6 PMC5029032 27650390

[34] Karen R. Catalase test protocol. Created: Thursday, 11 November 2010. Am Soc Microbiol 2016.

[35] Clinical and Laboratory Standard Institute (CLSI). : Performance Standards for Antimicrobial Susceptibility Testing; A CLSI Supplement for global application M100 2020 30th, 18–102.

[36] Deribe K. Podoconiosis today: challenges and opportunities. *Transactions of the Royal Society of Tropical Medicine and Hygiene* 2018, 112:473–5. 10.1093/trstmh/try087 PMC6214421 30137612

[37] Wanji S, Tendongfor N, Esum M, Che JN, Mand S, Tanga Mbi C, Eyong P, Hoerauf A (2008). Elephantiasis of non-filarial origin (podoconiosis) in the highlands of north–western Cameroon. Annals of Tropical Medicine & Parasitology.

[38] Grice EA, Segre JA (2011). The skin microbiome. Nat Rev Microbiol.

[39] Costello E, Lauber C, Hamady M, Fierer N, Gordon J, Knight R (2009). Bacterial community variation in human body habitats across space and time. Science.

[40] Beringer PM, Appleman MD (2000). Unusual respiratory bacterial flora in cystic fibrosis: Microbiology and clinical features. Curr opin pulm medicine.

[41] Wiersinga WJ, Virk HS, Torres GA, Currie BJ, Peacock SJ, Dance DAB, Limmathurotsakul D, Melodioidosis. Nat Reviews Disease Primers 2018, 4. 10.1038/nrdp.2017.107 PMC6456913 29388572

[42] Hauschild T, Schwarz S (2003). Differentiation of *Staphylococcus sciuri* strains isolated from free-living rodents and insectivores. J Vetenary Med Ser B.

[43] Stepanović S, Ježek P, Dakić I, Vuković D, Seifert L (2005). *Staphylococcus sciuri*: an unusual cause of pelvic inflammatory disease. Int J STD AIDS.

[44] Giordano N, Corallo C, Miracco C, Papakostas P, Montella A, Figura N, Nuti R (2012). Erythema nodosum associated with Staphylococcus xylosus septicemia. J Microbiol Immunol Infect.

[45] Shenoy RK, Sandhya K, Suma TK, Kumaraswami V (1995). A preliminary study of filariasis related acute adenolymphangitis with special reference to precipitating factors and treatment modalities. Southeast Asian Journal of Tropical Medicine Public Health.

[46] Rivera M, Diaz Dominguez M, Mendiola NR, Roso GR, Quereda C (2014). *Staphylococcus lentus* Peritonitis: a case report. J Int Soc Perit Dialysis.

[47] QuianY, Wei L, Siyue K, Xiaoping Y, Zhiqin G, Jlan G, Lianjuan Y. Epidemiology of interdidgital infections of toe web spaces in Shanghai, China: etiology, risk factors and therapeutic approaches. Res Square 2020, 6–10.

[48] Davey G, GebreHanna E, Adeyemo A, Rotimi C, Newport M, Desta K. Podoconiosis: a tropical model for gene–environment interactions? Trans R Soc Trop Med Hyg 2007, 101. 10.1016/j.trstmh.2006.05.002 16884751

[49] Shields BE, Tschetter AJ, Wanat KA (2016). *Staphylococcus simulans*: an emerging cutaneous pathogen. JAAD case reports.

[50] Heldt J, Papaloukas N, Timmernan CP (2019). A rare bloodstream infection: *Bacillus mycoides*. Netherland J Med.

[51] Addisu S, El-Metwally TH, Davey G, Worku Y, Titheradge MA. The role of transforming growth factor-beta1 and oxidative stress in podoconiosis pathogenesis. Bristish J Dermatology. May 2010;162(5):998–1003. 10.1111/j.1365-2133.2010.09652.x 20199540

[52] CDC. : Outbreak of cutaneous *Bacillus cereus* infections among cadets in a university military program viewed on the 05/06.2021 https://www.cdc.gov/mmwr/preview/mmwrhtml/mm5448a3.htm 2005. 16340940

[53] Borsa BA, Aldağ ME, Tunalı B, Dinç U, Güngördü Dalar Z, Özalp VC, Nadir GFP (2016). *Bacillus* a sepsis case caused by a rare opportunistic pathogen: *Bacillus pumilus*. Microbiol bulleting J.

[54] Bocchi CL, Perna A, Vitiello R, Greco T, Maccauro G, Perisano C. A rare case of Bacillus megaterium soft tissues infection. Acta bio-medica: Atenei Parmensis 2020, 9. 10.23750/abm.v91i14-S.10849 PMC7944702 33559642

[55] Kim R, Annette CR (2015). Mandell, Douglas, and Bennett’s principles and practice of Infectious Diseases. Other Coryneform Bacteria and Rhodococci.

[56] Heldt MLA, Cohen PR (2017). Staphylococcus lugdunensis infections of the skin and soft tissue: a Case Series and Review. Dermatology and therapy.

[57] Haydushka IA, Markova N, Kirina V, Atanassova M (2012). Recurrent sepsis due to *Bacillus licheniformis*. J global Infect Dis.

[58] Miki RA, Rubin LE, Kirks J, Doddis SD (2006). Spontaneous septic arthritis caused by *Burkholderia cepacia*. Lowa Orthop J.

[59] Cheong HS, Wi YM, Moon SY, Kang CI, Son JS, Ko KS, Ryeon D, Chung DR, Lee NY, Song JH, Peck KR (2008). Clinical features and treatment outcome of infection caused by. Sphingomonas paucimobilis Infectious control and hospital epidemiology.

[60] Isaacs RD, Paviour SD, Bunker DE, Lang SDR. Wound infection with aerogenic Aeromonas strains: a review of twenty-seven cases. Eur J Clin Microbiol Infect Dis 1988, 7. 10.1007/BF01962336 3137035

[61] Alwazzeh MJ, Alkuwaiti FA, Alqasim M, Alwartha S, El-ghoneimy Y (2020). Infective endocarditis cause by *Pseudomona stutzeri*; a case report and literature review. Infect disease Rep.

[62] Olszewski WL, Marzanna Z, Ewa S, Swoboda-Kopec E, Pradeep J, Karoon A, Sashi G, Arun G, Piotr A, Marek D. Cryptic Bacteria of Lower Limb Deep Tissues as a possible cause of inflammatory and necrotic changes in ischemia, venous stasis and Varices, and Lymphedema *Surgical infections* 2015, 16:313–22. 10.1089/sur.2014.019 PMC4487244 26046245

[63] National Center for Biotechnology Information. : PubChem Compound Summary for CID3467, Gentamycin. Retrieved June 13 /2021 from https://pubchem.ncbi.nlm.nih.gov/compound/Gentamycin 2021.

[64] Faulds TPA (1991). Ofloxacin. A reappraisal of its antimicrobial activity, Pharmacology and therapeutic use drugs. Springer Nat.

