# Supplementary material for: Phenotypical characterization, and antibiotics susceptibility patterns of skin bacteria found in podoconiosis patients in the North West Region of Cameroon
Source: BMC Microbiol. 2023 Jul 17;23:189. doi: 10.1186/s12866-023-02923-9 (PMC10351198; doi:10.1186/s12866-023-02923-9)
Supplement: Supplementary file 1 — Supplementary Material 1 [file 12866_2023_2923_MOESM1_ESM.docx]

| **Microbiome Analysis of Podoconiosis Patients in North West Region of Cameroon** | *Indiv. No*.: \|__\|__\|-\|__\|__\|__\|-\|__\|__\|__\| |
| --- | --- |
|  | *Date of visit*: \|__\|__\| / \|__\|__\| / \|__\|__\|__\|__\|  (dd/mm/yyyy) |

| 1. **DEMOGRAPHIC DATA** |
| --- |
| Gender:  female  male  Age (years): \|__\|__\|  Duration in the community: \|__\|__\|  (years)  Occupation (specify: ____________________________________)  Years of education: Zero (0)  1-7  8-15  16-22 |

| 1. **CLINICAL DATA** |
| --- |
| Lymphedema staging Left leg  Right Leg |

| 1. **MICROBIOME SAMPLING** | | | | |
| --- | --- | --- | --- | --- |
| **1** | Leg swabbing | | Date of visit: \|__\|__\| / \|__\|__\| / \|__\|__\|__\|__\|  (dd/mm/yyyy) | |
|  | |  |  | |
| **2.** Swabbed | | **Left** leg  yes  no | | **Right** Leg  yes  no |

| 1. **REMARKS** |
| --- |
|  |
